# Selective Depletion of Gut Gram-Negative Bacteria Attenuates Alcohol Binge-Induced Cardiovascular Dysfunction by Lowering Cardiac Anandamide Levels

**DOI:** 10.1016/j.ajpath.2025.10.010

**Published:** 2025-11-06

**Authors:** Sarah E. Cohen, Meagan E. Donovan, Davin J. Gardner, Danielle Sambo, Seray B. Karagoz, Resat Cinar, David Goldman, Jason D. Gardner, Pal Pacher, Janos Paloczi

**Affiliations:** ∗ Department of Physiology, Louisiana State University Health Sciences Center, New Orleans, Louisiana; † Laboratory of Neurogenetics, National Institute on Alcohol Abuse and Alcoholism, National Institues of Health, Bethesda, Maryland; ‡ Section on Fibrotic Disorders, National Institute on Alcohol Abuse and Alcoholism, National Institues of Health, Bethesda, Maryland; § Laboratory of Cardiovascular Physiology and Tissue Injury, National Institute on Alcohol Abuse and Alcoholism, National Institues of Health, Bethesda, Maryland

## Abstract

Binge drinking contributes to an increasing number of emergency department visits in the United States. Previous work demonstrated that an alcohol binge impairs cardiac performance and exerts complex hemodynamic effects through the activation of the endocannabinoid-mediated cannabinoid type 1 receptor (CB1R) signaling pathway. Anandamide (AEA), an endogenous CB1R agonist, is synthesized in response to various stressors and tissue injury. However, the role of binge drinking in increasing myocardial AEA levels, which leads to CB1R-dependent cardiodepression, remains unclear. This work studied how endotoxins from intestinal Gram-negative bacteria affect myocardial AEA levels, which further induce CB1R-dependent cardiac dysfunction following acute alcohol intoxication. Using a murine model of a single alcohol binge (5 g/kg orally), reduced mesenteric microcirculation concurrent with elevated circulating endotoxin levels was observed. Selective depletion of gut Gram-negative bacteria by antibiotics partially ameliorated alcohol-induced gut barrier dysfunction, significantly lowered circulating endotoxins, coinciding with reduced cardiac AEA levels at 3 hours after binge. These changes were paralleled with moderately improved cardiac performance and vascular tone. Cardiac RNA levels of genes involved in AEA synthesis increased after alcohol binge, but not in antibiotic-pretreated mice. However, acute alcohol-induced cardiac AEA formation was unrelated to toll-like receptor-4 signaling. These findings provide novel insights that highlight the pivotal role of intestinal Gram-negative bacteria in modulating cardiac AEA levels after an alcohol binge, leading to cardiovascular dysfunction.

## Graphical abstract

**Figure:**
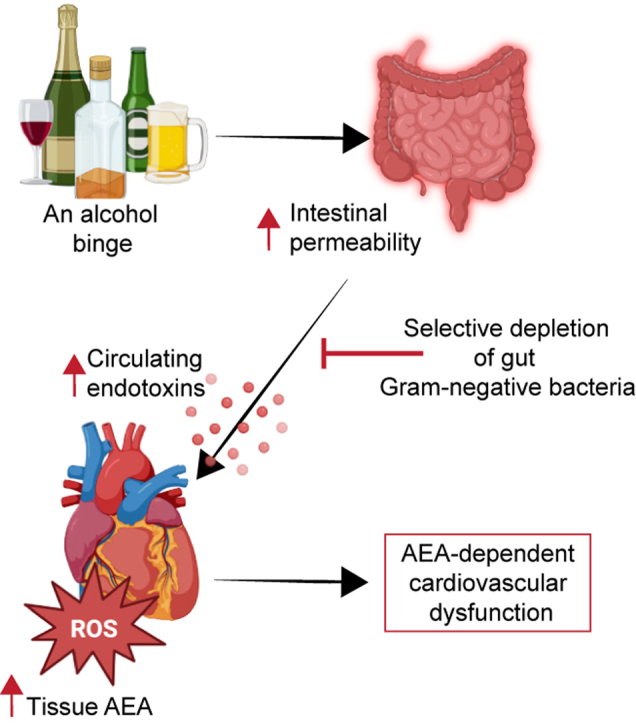

Alcohol remains the most commonly misused substance in the United States.^1^^,^^2^ According to the National Survey on Drug Use and Health, the prevalence of alcohol misuse has significantly increased in recent years.^3^ Recent epidemiologic data also reveal a worrisome increase in binge drinking, especially among young adults, leading to a significantly higher risk of health complications.^4^ According to the National Institute on Alcohol Abuse and Alcoholism, binge drinking is the consumption within a short period of time of five or more drinks by men and four or more drinks by women. A single episode of binge alcohol drinking has been associated with an increased risk of cardiovascular dysfunction, including tachyarrhythmias, hypotension, and inadequate tissue perfusion.^5^, ^6^, ^7^, ^8^, ^9^ Additionally, repeated episodes of excessive alcohol drinking may lead to cardiomyopathy and hypertension.^10^

Evidence also suggests that binge drinking leads to intestinal barrier dysfunction with consequences that may be pathogenic.^11^^,^^12^ Within hours of binge drinking, elevated serum endotoxin levels are detected in otherwise healthy individuals.^13^ Circulating endotoxins [mainly lipopolysaccharide (LPS)] are derived from intestinal Gram-negative bacteria. Alcohol-induced endotoxin translocation from the gastrointestinal tract to systemic circulation can promote inflammation, oxidative stress, and tissue damage across multiple organs, mainly through toll-like receptor (TLR) signaling pathways, including the activation of the TLR2 or TLR4 cascade.^14^, ^15^, ^16^, ^17^

Using a mouse model of acute alcohol administration, it was recently demonstrated that a single high dose of alcohol (3 to 5 g/kg) exerts complex and profound cardiovascular effects with transient but marked depression of myocardial contractility and cardiac output.^7^^,^^18^ These adverse cardiovascular effects of an alcohol binge involve the activation of the cannabinoid type 1 receptor (CB1R) signaling axis, leading to CB1R-dependent cardiodepression and blood redistribution.^7^ It has also been shown that the activation of CB1R signaling in the cardiovascular system, by either the endocannabinoid anandamide (AEA) or exogenously administered CB1R agonists, negatively affects cardiac performance and vascular tone regulation.^18^, ^19^, ^20^, ^21^, ^22^, ^23^, ^24^ In contrast, the activation of the cannabinoid type 2 receptor does not affect cardiac contractility.^7^^,^^25^^,^^26^ Previous research also indicated that blocking CB1R activation following alcohol intoxication with i.v. rimonabant, a prototypical CB1R antagonist/inverse agonist, can partially restore acute alcohol-induced cardiac dysfunction and peripheral vasodilation, suggesting a potential therapeutic avenue in acute alcohol intoxication.^7^

The formation of tissue endocannabinoids is tightly regulated by physiological and pathologic triggers. Cellular stressors, including oxidative stress, inflammation, and metabolic disturbances, are key inducers of AEA synthesis in both the liver and the cardiovascular system.^26^^,^^27^ Furthermore, LPS has also been shown to trigger AEA synthesis in different model systems, contributing to organ dysfunction and injury.^28^, ^29^, ^30^ However, the implications of elevated serum LPS following alcohol binge drinking for cardiac AEA levels remain unclear. Therefore, this study aimed to investigate the relationship between the acute alcohol-induced increase in circulating endotoxin levels and cardiac AEA levels, which promote CB1R-dependent cardiodepression and blood redistribution.

## Materials and Methods

### Animals

Animal handling and used were in accordance with NIH and Louisiana State University Health Sciences Center (New Orleans, LA) guidelines and protocols, as approved by the respective Institutional Animal Care and Use Committees. For this study, 8- to 12-week–old male and female C57BL/6J mice were obtained from The Jackson Laboratory (Bar Harbor, ME). Male and female toll-like receptor-4 knockout (*Tlr4*^*−/−*^) mice on the C57BL/6J background, along with their corresponding wild-type controls, were also used in this study and obtained from The Jackson Laboratory (strain number 029015).

### Experimental Design

In the first set of experiments, mice were exposed to a single alcohol gavage at a dose of 5 g/kg body weight after overnight fasting. Control mice were similarly administered normal saline. Intestinal microcirculation and levels of systemic circulating endotoxins were evaluated at different time points following alcohol gavage. In the next set of experiments, mice were treated orally with a mixture of antibiotics (Sigma-Aldrich, St. Louis, MO) before saline or alcohol gavage to selectively eliminate intestinal Gram-negative flora, as previously described.^31^^,^^32^ Briefly, mice were randomly assigned to control or antibiotic treatment groups, where they received either water or nonabsorbable antibiotics (neomycin, polymyxin-B, and nystatin; 200, 150, and 1 mg/kg, respectively) for a 14-day pretreatment period before saline or alcohol gavage. After the pretreatment, mice were administered saline or alcohol orally, as described above. On the basis of previous findings, end points were collected 3 hours after alcohol gavage.^7^ In a separate set of experiments, *Tlr4*^*–/–*^ mice and their wild-type controls were also given saline or alcohol orally. End points were collected 3 hours after oral administration of saline and alcohol.

### Pressure-Volume Catheterization

Left ventricle (LV) performance was assessed using a pressure-volume approach. Briefly, a pressure-volume catheter equipped with a 1.4-F size microtip (SPR-839; ADInstruments, Colorado Springs, CO) was introduced into the right carotid artery and advanced into the LV. After stabilization, pressure-volume signals were continuously recorded using the PowerLab LabChart data acquisition system (ADInstruments). Multiple load-dependent and load-independent LV function measures were evaluated, including the maximal slope of pressure increment (dP/dt_max_), end-systolic pressure, stroke work, stroke volume, cardiac output, ejection fraction, end-systolic elastance, preload-recruitable stroke work, and the relationship between dP/dt_max_ and end-diastolic volume. Vascular indexes, such as mean arterial blood pressure and total peripheral resistance, were also assessed as described previously.^7^^,^^33^

### Arterial and Microvascular Blood Perfusion Measurements

The blood flow in the superior mesenteric arteries was recorded using perivascular transonic Doppler flow probes (MA0.7PSB; ADInstruments) and evaluated using PowerLab LabChart (ADInstruments). The rate of microcirculation in the hind limbs and mesenteries was also assessed using a moorFLPI-2 laser speckle contrast imaging system (Moor Instruments, Wilmington, DE).^7^^,^^34^^,^^35^

### Tissue Endocannabinoid Measurement

Levels of anandamide (AEA) and 2-arachidonoyl glycerol (2-AG) in the myocardium were measured using liquid chromatography coupled with inline mass spectrometry (Agilent Technologies, Santa Clara, CA).^7^^,^^21^

### Serum Alcohol Measurement

Blood samples were collected from the inferior vena cava of terminally anesthetized mice. Serum alcohol levels were then determined using the AM1 Alcohol Analyzer (Analox Instruments, Stourbridge, UK).^36^

### Serum Lipopolysaccharide Measurement

Serum endotoxin (LPS) levels were measured using the Pierce LAL Chromogenic Endotoxin Quantitation Kit, according to the manufacturer's instructions (Thermo Fisher Scientific, Waltham, MA).

### Serum Alanine Aminotransferase and Blood Urea Nitrogen Measurement

Serum alanine aminotransferase activity and blood urea nitrogen levels were measured to determine liver and kidney damage, respectively, using a clinical chemistry analyzer system (VetTest 8008; IDEXX Laboratories, Westbrook, ME).

### Intestinal Permeability Measurement

Fluorescein isothiocyanate–dextran (4 kDa; Sigma-Aldrich) was freshly dissolved in phosphate-buffered saline and orally administered to the mice (600 mg/kg) 1 hour before the experimental end point. Portal blood was collected by venipuncture at 3 hours after the oral administration of saline or alcohol. Plasma samples were then diluted in phosphate-buffered saline, and fluorescence was measured by using a plate reader (495-nm emission/520-nm excitation; Molecular Devices, San Jose, CA).

### Cardiac Tissue RNA Isolation and AmpliSeq

Cardiac tissues were homogenized in TRIzol (Invitrogen, Carlsbad, CA), and total RNA was isolated with a Direct-zol RNA Miniprep Kit (Zymo Research, Irvine, CA), according to the manufacturer's instructions. RNA concentrations were determined using a NanoDrop 2000 Spectrophotometer (Thermo Fisher Scientific). AmpliSeq libraries were prepared using the Ion AmpliSeq Library Kit Plus (Thermo Fisher Scientific; 4488990), per the manufacturer's instructions. Library quality was determined using the Agilent High Sensitivity DNA Kit (Agilent Technologies; 5067-4626), and library concentrations were quantified using the Ion Library TaqMan Quantitation Kit (Life Technologies, Carlsbad, CA; 4468802), per manufacturer's instructions. Barcoded AmpliSeq libraries were loaded onto the sequencing chips using the Ion 540 Chip Kit (Thermo Fisher Scientific; A27766) and Ion Chef Instrument. Libraries were sequenced on the Ion Torrent S5 Sequencing System via the Ion Torrent 540-OT2 kit (Thermo Fisher Scientific; A27753). Eight to nine libraries were loaded per sequencing chip, and two sequencing chips were loaded and sequenced at a time. An average of 8.5 million counts per sample were sequenced. Alignment and gene expression count were performed using Ion Torrent AmpliSeq RNA Plugin version 0.5.4.0 (Thermo Fisher Scientific) using the mm10 genome as a reference. The raw RNA-sequencing files are released and available to the public through the Gene Expression Omnibus database (*https://www.ncbi.nlm.nih.gov/geo*; accession number GSE294937). Raw RNA counts and metadata are also provided in Supplemental Table S1.

### Quantitative Real-Time PCR

Equal amount of RNAs (1 μg), isolated from cardiac tissues, was reverse transcribed using High-Capacity cDNA Reverse Transcription Kit with RNAse Inhibitor (Thermo Fisher Scientific), according to the manufacturer's instructions. Quantitative real-time PCR was performed using gene-specific primers and SYBR Select Master Mix (Thermo Fisher Scientific) on a CFX Opus 96 real-time PCR platform (Bio-Rad, Hercules, CA). The sequences of the custom-designed primer pairs (Thermo Fisher Scientific) were as follows: *Actb* (accession number NM_007393.3), forward 5′-CACCCGCGAGCACAGCTTCTT-3′ and reverse 5′-TTTGCACATGCCGGAGCCGTT-3′; *Napepld* (accession number NM_001359964.1), forward 5′-ATGGCTGATAATGGAGAAGAATCAC-3′ and reverse 5′-CGTCTTCAGGGTCACTGACAAA-3′; *Gde1* (accession number NM_019580.4), forward 5′- CTCCGCCATAACCTCACCAT-3′ and reverse 5′-TTCCGGCAAGAATGAGCAGA*-*3′; *Abdh4* (accession number NM_134076.3), forward 5′-CGGCAGGGCTTGTTTACTAT-3′ and reverse 5′-TCTCCCGCCATGTCTCTATT*-*3′; and *Faah* (accession number NM_010173.6), forward 5′-GGGTGTGACATCGGAGAGTG-3′ and reverse 5′-CGGTTCCCAGTAGGCTTGAG*-*3′. Cycling conditions comprised initial polymerase activation at 95°C for 2 minutes, followed by 40 cycles of 95°C for 15 seconds and 60°C for 1 minute. Amplification specificity was confirmed by melting curve analysis of the PCR products. Quantification was performed using the comparative cycle threshold (C_T_) method (2^–ΔC_T_^ was normalized to the housekeeping gene. The primer pairs used in this study match their corresponding RNA sequences that are publicly available through their specific accession numbers (*https://www.ncbi.nlm.nih.gov/nuccore*).

### 16S rRNA Sequencing

Amplicon libraries targeting the bacterial 16S rRNA gene variable regions (V1 to V8) were constructed using a two-step PCR protocol by the supplier (Microbiome Insights, Richmond, BC, Canada). Briefly, in the first PCR step, up to 25 ng of extracted DNA was used as the template. The cycling conditions for PCR were as follows: initial denaturation at 98°C for 3 minutes, followed by 30 amplification cycles (98°C for 30 seconds, 62°C for 30 seconds, and 72°C for 2 minutes), concluding with a final elongation step at 72°C for 5 minutes. The primers used for amplification flanked the 16S rRNA V1 to V8 region: the forward primer (27F) was 5′-AGRRTTYGATYHTDGYTYAG-3′, and the reverse primer (1391R) was 5′-GACGGGCGGTGWGTRCA-3’. In the second PCR step, PCR barcoding expansion 1-96 (EXP-PBC096) was used to uniquely tag each sample with 24-nucleotide barcode sequences, in accordance with the manufacturer's protocol. The resulting barcoded amplicon libraries were pooled and purified using Illumina (San Diego, CA) Purification Beads. Gel size selection was performed to isolate DNA fragments ranging from 1000 to 2000 bp. Sequencing libraries were subsequently prepared from these purified amplicons using the SQK-LSK114 kit (Oxford Nanopore Technologies, Oxford, UK), adhering to the manufacturer's instructions, with an input of 35 to 50 fmol of DNA. DNA concentration was quantified using the Qubit dsDNA HS Assay kit (Thermo Fisher Scientific).

The prepared sequencing library was loaded onto a MinION Mk1B MIN-101B Flowcell (R10.4.1; FLO-MIN114) and sequenced using MinKNOW software version 24.06.14 (Oxford Nanopore Technologies). Basecalling and demultiplexing were performed with MinKNOW Dorado 7.4.13 using the superaccurate basecalling model version 4.3.0 (configuration r10.4.1_400 bps_sup.cfg) and custom barcode sequences. Sequencing reads were demultiplexed and filtered for length (320 to 2000 bp) and quality (Phred score >15). Filtered reads were mapped with Emu version 3.5.1 to its default database (a combination of rrnDB version 5.6 and National Center for Biotechnology Information 16S RefSeq; containing 49,301 sequences from 17,555 unique bacterial and archaeal species). Raw 16S rRNA counts and metadata are included in Supplemental Table S2.

### Histologic Analysis

Tissue sections were prepared for histology as previously described.^34^^,^^36^ After routine formalin-fixed, paraffin-embedded specimen processing, tissue sections (4 μm thick) were prepared and stained. For immunohistochemistry, deparaffinized sections underwent heat-mediated antigen retrieval (pH = 6 citrate buffer, at 95°C for 15 minutes; Electron Microscopy Science, Hatfield, PA). Subsequently, sections were incubated in BLOXALL solution (Vector Laboratories, Burlingame, CA) to block endogenous peroxidase activity, according to the manufacturer's instructions. Sections were then incubated with anti–4-hydroxinonenal (4-HNE) primary antibodies (JaICA, Nikken SEIL Co, Lt., Fukuroi, Shizuoka, Japan; number MHN-020P; at the dilution of 1:200) overnight at 4°C in a humidified chamber. Myocardial sections were then incubated with an anti-mouse IgG conjugated with a horseradish-peroxidase polymer (Mouse-On-Mouse Detection Kit; Vector Laboratories), according to the kit's instructions. Color development was induced by incubation with a diaminobenzidine chromogenic substrate (Vector Laboratories) for 60 to 90 seconds, and the sections were counterstained with hematoxylin. Finally, the sections were dehydrated in ethanol, and cleared in xylene before mounting. Images were captured using a microscope set (Olympus, Center Valley, PA). Immunolabeling was performed in four to six heart sections per group, and five to seven random areas were captured. Images were analyzed using ImageJ2 software version 2.14.0/1.54f (NIH, Bethesda, MD; *https://downloads.imagej.net/fiji/releases/2.14.0*).

### 4-HNE Enzyme-Linked Immunosorbent Assay

Cardiac tissue 4-HNE levels were quantified using a competitive enzyme-linked immunosorbent assay kit, according to the manufacturer's instructions (Cell Biolabs, San Diego, CA; number STA-838).^36^

### Statistical Analysis

Data are expressed as means ± SEM. Statistical significance between groups was determined by *t*-test or, in case of multiple comparisons, two-way analysis of variance, followed by Bonferroni *post hoc* test to compare select groups with their corresponding controls. Equality of variances was also examined using either the F-test (for comparing two groups only) or D'Agostino-Pearson omnibus, Anderson-Darling, Shapiro-Wilk, and Kolmogorov-Smirnov tests (for multiple comparisons). The analysis was performed using GraphPad Prism software version 10.6.1 (GraphPad Software, San Diego, CA), except for the cardiac RNA sequencing data set, where the open-source R version 4.4.2 (DESeq2 package) and g:Profiler software version e113_(eg, 59_p19_f6a03c19) were used for differential gene expression and functional enrichment analysis, respectively.^37^^,^^38^ Probability values of *P* < 0.05 were considered significant.

Statistical analysis of the 16S rRNA sequencing data was performed using the maaslin3 package in R to identify microbial taxa associated with treatment. Adjusted *P* values <0.1 were considered to indicate a statistically significant difference.

## Results

### A Single Alcohol Binge Reduces the Rate of Mesenteric Blood Perfusion, which Coincides with an Increase in Systemic Endotoxin Levels

**Figure 1:**
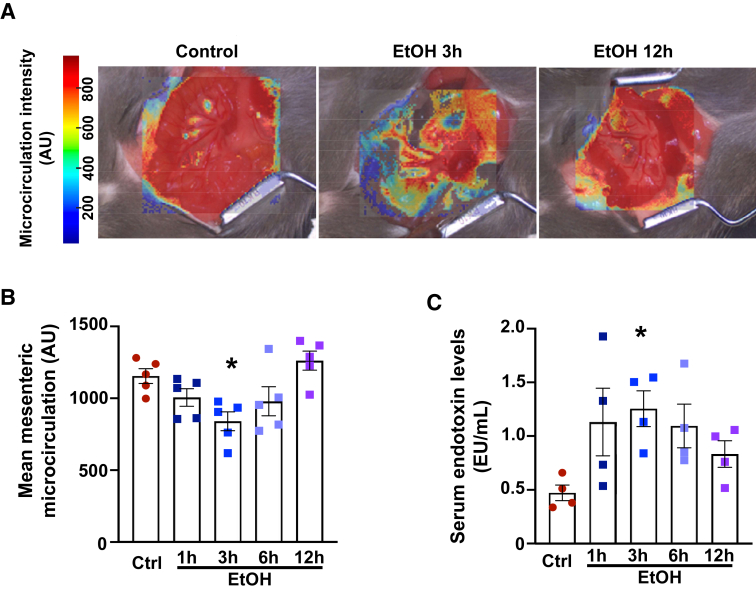
Alcohol binge-induced vascular dysfunction and blood redistribution impair mesenteric microcirculation, leading to endotoxemia. **A:** Representative images of the intestinal microcirculation in control and alcohol-binged mice 3 and 12 hours after a single alcohol gavage. Red indicates higher intensity, whereas blue represents the lower rate of microcirculation. **B:** The results of microcirculation in control (ctrl) and alcohol [ethanol (EtOH)]–gavaged mice at different time points following the alcohol gavage. **C:** Serum levels of endotoxin in ctrl and alcohol (ethanol)–gavaged mice at different time points following the alcohol gavage. Data are presented as individual values with group means ± SEM (**B** and **C**). *n* = 4 to 5 (**B** and **C**). ∗*P* < 0.05 versus ctrl group (one-way analysis of variance, Dunnett multiple comparison test). AU, arbitrary unit; EU, endotoxin unit.

First, to study the effects of an alcohol binge on mesenteric blood perfusion, mice were gavaged once with 5 g/kg alcohol or saline. Intestinal microcirculation was assessed by laser speckle contrast imaging at 1, 3, 6, and 12 hours following the gavage. A significant reduction in the rate of intestinal microcirculation in mice 3 hours after the alcohol gavage was observed, which recovered within 12 hours (Figure 1, A and B). Simultaneously, increased systemic serum levels of endotoxin were documented (Figure 1C), paralleling the peak decrease in intestinal blood perfusion.

### Selective Depletion of Gut Gram-Negative Bacteria Lowers Circulating Endotoxin Levels and Partially Ameliorates Intestinal Barrier Dysfunction following Acute Alcohol Exposure

**Figure 2:**
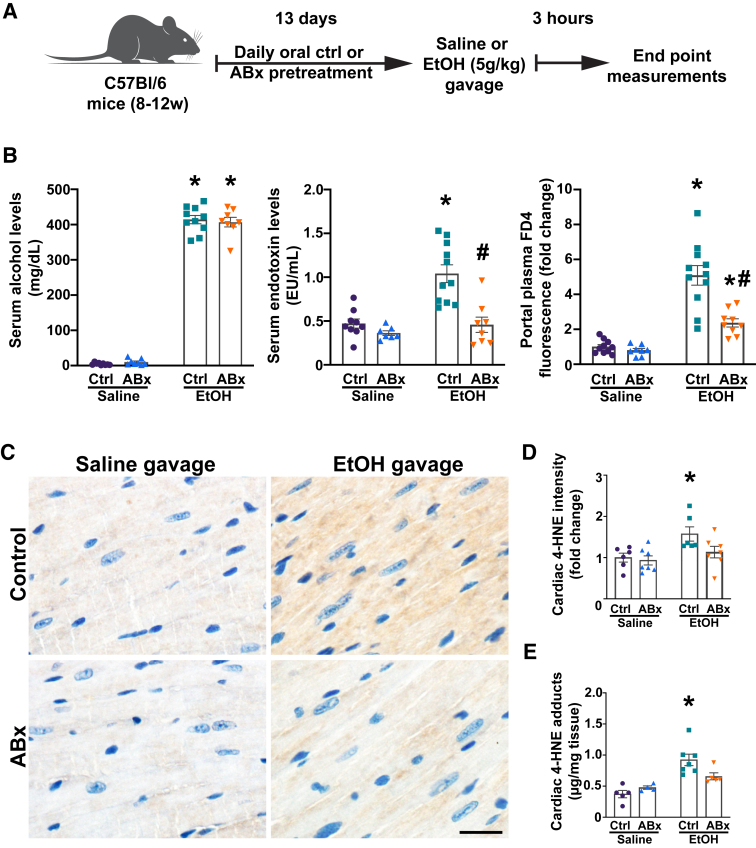
Selective depletion of gut Gram-negative bacteria reduces circulating endotoxin levels and partially ameliorates gut barrier dysfunction following an alcohol binge. **A:** Study design and groups. **B:** Serum alcohol endotoxin, and portal fluorescein isothiocyanate–dextran-4 (FD4) levels measured 3 hours after administration of saline or alcohol [ethanol (EtOH)] in mice, with or without antibiotic pretreatment (ABx or ctrl, respectively). **C:** Representative images of 4-hydroxynonenal (4-HNE) immunostaining of cardiac sections from saline or alcohol (ethanol)–gavaged mice with or without antibiotic pretreatment (ABx or ctrl, respectively). **D:** Semiquantitative assessment of 4-HNE adducts in cardiac sections of saline or alcohol (ethanol)–gavaged mice, with or without antibiotic pretreatment (ABx or ctrl, respectively). **E:** Quantitative assessment of 4-HNE adducts in cardiac homogenates from saline or alcohol (ethanol)–gavaged mice, with or without antibiotic pretreatment (ABx or ctrl, respectively). Data are presented as individual values with group means ± SEM (**B**, **D**, and **E**). *n* = 7 to 11 (**B**); *n* = 4 to 7 (**E**). ∗*P* < 0.05 versus corresponding saline group; ^#^*P* < 0.05 versus ctrl ethanol group (two-way analysis of variance, Bonferroni multiple comparison test). Scale bar = 50 μm (**C**). EU, endotoxin unit.

To assess the role of alcohol-induced increased systemic endotoxin levels on the cardiovascular system, a mixture of nonabsorbable antibiotics (ABx) was used to selectively deplete intestinal Gram-negative bacteria in mice before alcohol gavage (Figure 2A). Although a significant decrease in body weight was documented throughout the ABx pretreatment, no difference was found in serum liver and kidney damage markers in the ABx-pretreated groups compared with controls (Supplemental Figure S1, A and B). The successful depletion of intestinal Gram-negative bacteria following ABx pretreatment was confirmed using 16S rRNA gene sequencing analysis of fecal samples, with fecal bacterial community composition assessed before and after antibiotic administration (Supplemental Figure S1C).

Blood alcohol levels in the control mice were comparable to those in the ABx-pretreated cohort (Figure 2B), as measured 3 hours after the gavage. However, systemic circulating endotoxin levels were significantly lower in the ABx-pretreated group 3 hours after acute alcohol exposure (Figure 2B). ABx pretreatment partially ameliorated acute alcohol-induced intestinal barrier dysfunction, as shown by decreased fluorescein isothiocyanate–dextran-4 translocation into the mesenteric circulation (Figure 2B). Furthermore, acute alcohol exposure significantly increased myocardial levels of the lipid peroxidation marker 4-HNE, whereas ABx pretreatment only showed a trend toward reduced cardiac 4-HNE adduct formation, as assessed by immunolabeling and competitive enzyme-linked immunosorbent assay. Notably, no difference was found among the saline controls.

### Cardiac Bulk RNA Sequencing Reveals Marked Changes in the Gene Expression Induced by an Alcohol Binge

To further evaluate the myocardial effects of selective elimination of gut Gram-negative microbiota before alcohol gavage, transcriptional differences in cardiac tissue between these four groups were examined. Acute alcohol intoxication induced marked cardiac gene expression changes that were not observed in the ABx-pretreated groups: the gene expression patterns among the control cohorts showed 1120 differentially expressed genes following alcohol exposure compared with the saline-gavaged group, whereas alcohol exposure resulted in significantly fewer (110) differentially expressed genes in the hearts with ABx pretreatment compared with the corresponding ABx saline control group (Supplemental Figures 1A and 2B). Notably, ABx pretreatment resulted in minimal changes in heart gene expression, as indicated by comparing the saline-gavaged groups (Supplemental Figure 2B).

**Figure 3:**
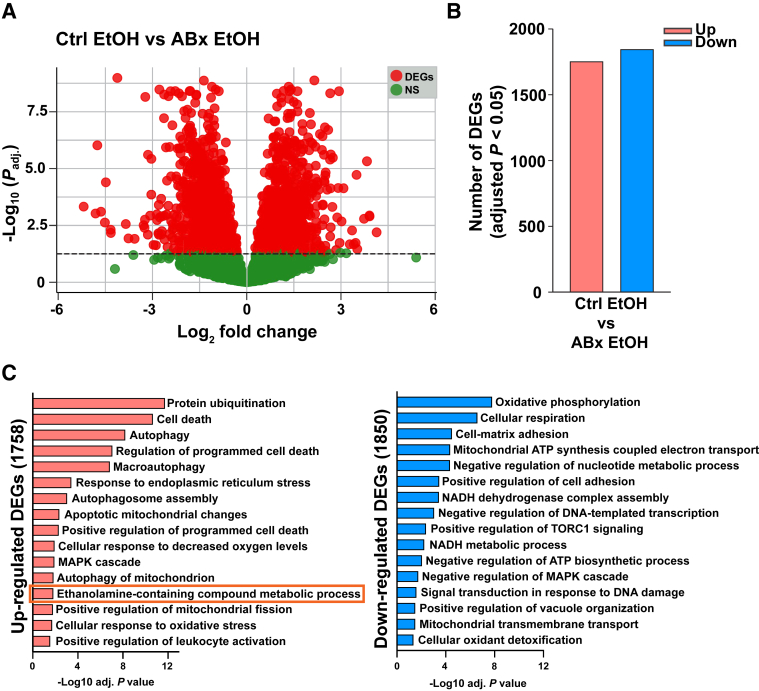
Alcohol binge-induced changes in the cardiac gene expression landscape in control (ctrl) mice compared with the antibiotic (ABx)–pretreated group. **A:** Volcano plots of the cardiac transcriptome comparing the control alcohol [ctrl ethanol (EtOH)] group with the antibiotic-pretreated alcohol (ABx ethanol) group. The red color indicates differentially expressed genes (DEGs) in the myocardium (adjusted *P* < 0.05), and the green color shows nonsignificant changes. **Horizontal dashed line** represents the threshold for statistical significance (log10_adjusted *P*_). **B:** The number of myocardial DEGs (adjusted *P* < 0.05) showing transcriptional differences between alcohol (ethanol) gavaged mice, with or without antibiotic pretreatment (ABx or ctrl, respectively). **C:** Significant Gene Ontology biological process pathways (adjusted *P* < 0.05) associated with the up-regulated and down-regulated DEGs in the ctrl-pretreated alcohol (ethanol)–gavaged group compared with the antibiotic (ABx)–pretreated alcohol (ethanol)–gavaged group. MAPK, mitogen-activated protein kinase; TORC1, target of rapamycin complex 1.

To evaluate the impact of ABx pretreatment on myocardial gene expression patterns following an alcohol binge, the transcriptome profiles of the two alcohol-exposed groups were compared as well. Acute alcohol exposure resulted in the up-regulation of 1758 genes and the down-regulation of 1850 genes relative to the ABx-pretreated alcohol gavage group (Figure 3, A and B). Subsequently, a functional analysis was performed using Gene Ontology biological processes to reveal altered functions based on changes in the transcriptome (Figure 3C). It was found that apoptotic and cell death processes were up-regulated along with cellular stress response, autophagy, mitogen-activated protein kinase cascade, and protein ubiquitination in the control alcohol-exposed group compared with the ABx-pretreated alcohol-exposed group (Figure 3C). Similarly, functions related to ethanolamide metabolic processes were also significantly up-regulated in the control alcohol-binged group. Processes associated with ATP synthesis and NADH metabolism were significantly down-regulated in the control alcohol-exposed group compared with the ABx alcohol-gavaged group (Figure 3C).

**Figure 4:**
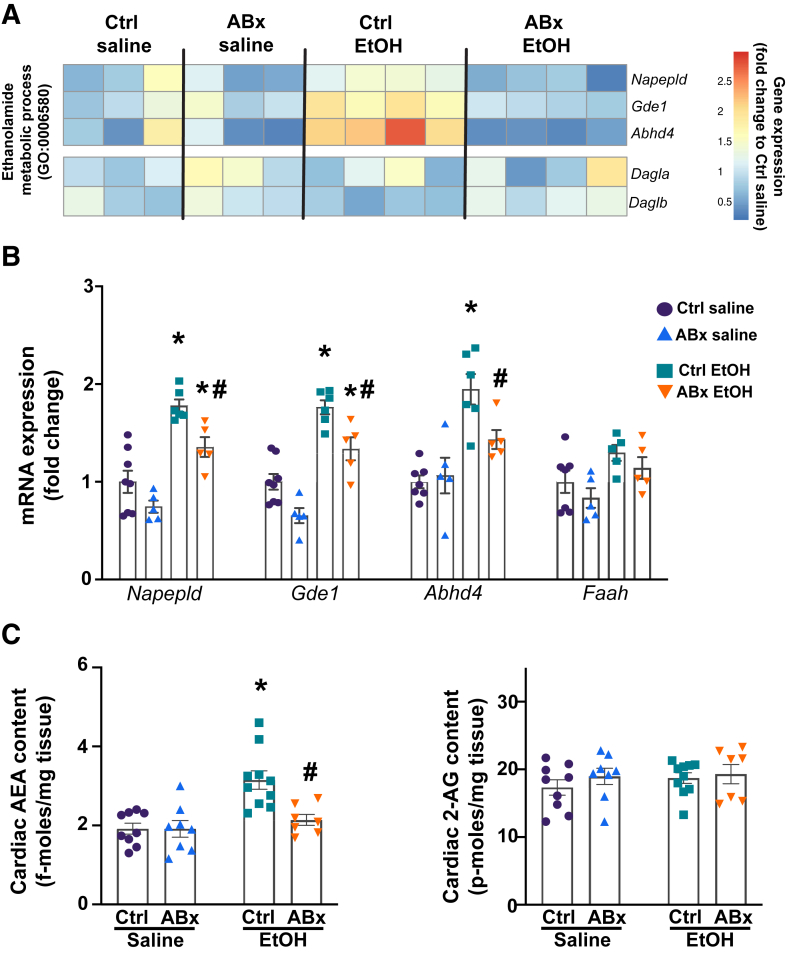
A single alcohol binge significantly increases myocardial levels of anandamide (AEA) in the control (ctrl)–pretreated group but not in the antibiotic (ABx)–pretreated group. **A:** Heat map of cardiac gene expression (fold change relative to the control saline group) for genes involved in ethanolamide metabolic pathways [Gene Ontology (GO):0006580, *https://biit.cs.ut.ee/gprofiler/gost*, last accessed November 1, 2025]. **B:** Cardiac mRNA levels of genes involved in anandamide synthesis (*Napepld*, *Gde1*, and *Abhd4*) and degradation (*Faah*) in mice gavaged with either saline or alcohol [ethanol (EtOH)], with or without antibiotic pretreatment (ABx or control, respectively). **C:** Myocardial levels of AEA and 2-arachidonoyl glycerol (2-AG), 3 hours after the administration of saline or alcohol (ethanol), with or without antibiotic pretreatment (ABx or ctrl, respectively). Data are presented as individual values with group means ± SEM (**B** and **C**). *n* = 5 to 10 (**B** and **C**). ∗*P* < 0.05 versus corresponding saline group; ^#^*P* < 0.05 versus ctrl ethanol group (two-way analysis of variance, Bonferroni multiple comparison test).

Focusing specifically on differentially expressed genes involved in the ethanolamide biosynthesis, elevated expression of genes involved in the synthesis of the endocannabinoid AEA (eg, *Gde1*, *Abhd4*) was found. In contrast, for the other major endocannabinoid, 2-AG, no changes in the levels of *Dagla* or *Daglb* was found across the groups (Figure 4A). To validate the results of cardiac bulk RNA sequencing, the cardiac levels of genes involved in the metabolism of AEA was assessed, including *Napepld*, *Gde1*, *Abhd4*, and *Faah*, using quantitative real-time PCR. A significant increase in the expression of AEA synthetic genes (*Napepld*, *Gde1*, *Abhd4*) was observed in the myocardium after acute alcohol exposure; however, ABx pretreatment partially reduced these gene expression levels following alcohol intoxication (Figure 4B), whereas the mRNA levels of the major AEA catabolic gene *Faah* remained unchanged across the groups.

Also, myocardial levels of endocannabinoids AEA and 2-AG were assessed in the myocardium using tandem liquid chromatography/mass spectrometry (Figure 4C). Significantly elevated myocardial AEA levels were found 3 hours after alcohol gavage compared with control hearts. Selective depletion of gut Gram-negative bacteria attenuated the acute alcohol-induced increase in cardiac AEA. In contrast, 2-AG levels remained unchanged across the groups (Figure 4C).

### Decreased Cardiac Anandamide Levels Resulting from Gram-Negative Bacteria Depletion from the Gut Attenuate Binge Alcohol-Induced Anandamide Formation and Subsequent Cardiac Dysfunction, Peripheral Vasodilation, and Blood Redistribution

**Figure 5:**
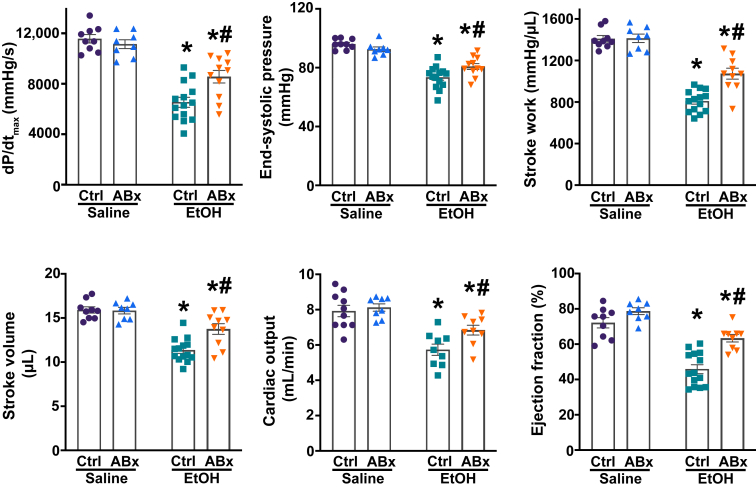
Selective depletion of gut Gram-negative bacteria attenuates binge alcohol-induced adverse cardiac effects. Systolic indexes of left ventricular performance [maximal slope of pressure increment (dP/dt_max_), end-systolic pressure, stroke work, stroke volume, cardiac output, and ejection fraction] in mice 3 hours after the administration of saline or alcohol [ethanol (EtOH)], with or without antibiotic pretreatment (ABx or ctrl, respectively). Data are presented as individual values with group means ± SEM. *n* = 8 to 14. ∗*P* < 0.05 versus corresponding saline group; ^#^*P* < 0.05 versus ctrl ethanol group (two-way analysis of variance, Bonferroni multiple comparison test).

To assess cardiovascular function following acute alcohol exposure with or without ABx pretreatment, pressure-volume catheterization was used 3 hours after a single alcohol or saline gavage (Figure 5). It was found that acute alcohol intoxication caused a significant cardiodepression, as indicated by the major LV systolic indexes, including the maximal rate of LV pressure increase (dP/dt_max_), end-systolic pressure, stroke work, stroke volume, cardiac output, and ejection fraction, compared with their corresponding control. In contrast, ABx-pretreated mice exhibited significantly better LV systolic performance following acute alcohol administration by the same metrics.

**Figure 6:**
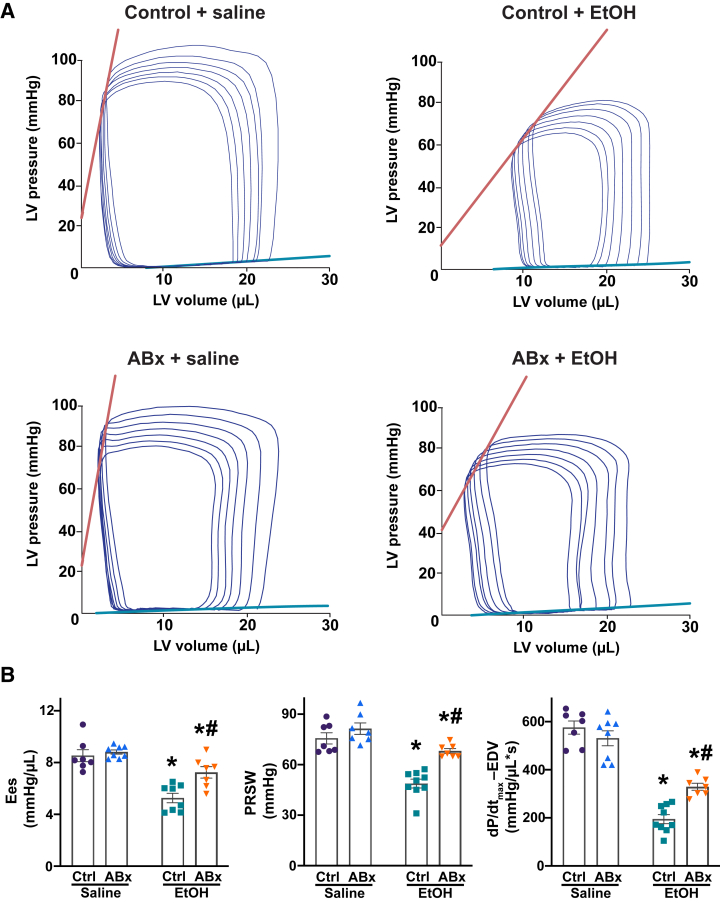
Selective depletion of gut Gram-negative bacteria mitigates binge alcohol-induced depression of load- and heart rate–independent indexes of left ventricular (LV) contractile function. **A:** Representative pressure-volume (P-V) loops during gradual preload reduction obtained by vena cava occlusion in mice 3 hours after the administration of saline or alcohol [ethanol (EtOH)], with or without antibiotic pretreatment (ABx or ctrl, respectively). **Red lines** indicate the slope of the end-systolic P-V relationship, whereas **green lines** depict the slope of the end-diastolic P-V relationship. **B:** Load- and heart rate–independent indexes of LV performance [end-systolic elastance (Ees), preload-recruitable stroke work (PRSW), and the dP/dt_max_–end-diastolic volume (dP/dt_max_-EDV) relation] in the saline or alcohol (ethanol)–gavaged groups, with or without antibiotic pretreatment (ABx or ctrl, respectively). Data are presented as individual values with group means ± SEM (**B**). *n* = 7 to 9 (**B**). ∗*P* < 0.05 versus corresponding saline group; ^#^*P* < 0.05 versus ctrl ethanol group (two-way analysis of variance, Bonferroni multiple comparison test).

Furthermore, it was found that the intrinsic contractility of the LV was significantly impaired by acute alcohol administration, as indicated by load- and heart rate–independent indexes of LV performance (namely, end-systolic elastance, preload-recruitable stroke work, and the dP/dt_max_–end-diastolic volume relationship) (Figure 6). Selective depletion of intestinal Gram-negative bacteria mitigated acute alcohol-induced reduction of these intrinsic contractile parameters.

**Figure 7:**
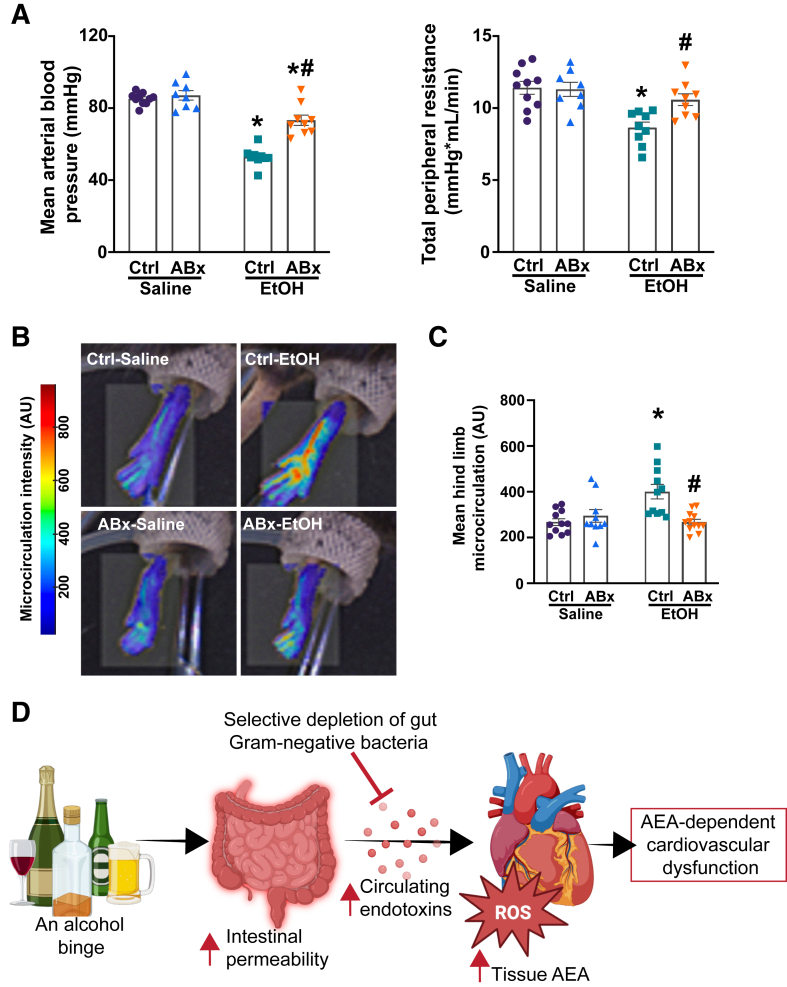
Selective depletion of gut Gram-negative bacteria mitigates binge alcohol-induced peripheral vasodilation and blood redistribution. **A:** Mean arterial blood pressure and total peripheral resistance measured in mice 3 hours after the administration of saline or alcohol [ethanol (EtOH)], with or without antibiotic pretreatment (ABx or ctrl, respectively). **B:** Representative images of the hind limb microcirculation in the saline or alcohol (ethanol)–gavaged groups, with or without antibiotic pretreatment (ABx or ctrl, respectively). Red indicates higher intensity, whereas blue represents lower rate of microcirculation. **C:** The results of hind limb microcirculation in mice 3 hours after the gavage saline or alcohol (ethanol) gavage, with or without antibiotic pretreatment (ABx or ctrl, respectively). **D:** Selective depletion of intestinal Gram-negative flora reduces circulating endotoxin levels following an alcohol binge, decreases tissue anandamide (AEA) levels, and improves cardiovascular function. Data are presented as individual values with group means ± SEM (**A** and **C**). *n* = 8 to 10 (**A**); *n* = 8 to 12 (**C**). ∗*P* < 0.05 versus corresponding saline group; ^#^*P* < 0.05 versus ctrl ethanol group (two-way analysis of variance, Bonferroni multiple comparison test). AU, arbitrary unit; ROS, reactive oxygen species.

Additionally, alcohol binge induced a decrease in mean arterial blood pressure and total peripheral resistance in mice 3 hours after gavage (Figure 7A), which resulted from decreased cardiac function (Figures 5 and 6) and dramatic redistribution of circulation, as shown by hind limb vasodilation (Figure 7, B and C). In contrast, the selective depletion of Gram-negative bacteria from the gut before alcohol gavage significantly mitigated alcohol-induced peripheral vasodilation, as indicated by higher mean arterial blood pressure, increased total peripheral resistance, and a corresponding decrease in hind limb vasodilation (Figure 7, A–C). Conversely, no difference was found in intestinal microcirculation among the alcohol-gavaged groups (Supplemental Figure 3, A and B), although the blood flow through the superior mesenteric artery was significantly higher in the ABx-pretreated group following an alcohol binge (Supplemental Figure 3C). Notably, no difference was found in LV contractile and vascular parameters among the control groups.

### Mice Lacking Toll-Like Receptor-4 Are Not Resistant to Binge Alcohol-Induced Cardiac Anandamide Formation and Subsequent Contractile Dysfunction

To investigate the molecular mechanisms behind endotoxin-induced myocardial AEA formation after acute alcohol intoxication, mice lacking *Tlr4* and their wild-type counterparts were exposed to saline and alcohol gavage. Despite the similar high serum alcohol and increased serum endotoxin levels, myocardial tissue levels of AEA were also elevated in *Tlr4* knockout mice following acute ethanol exposure, similarly to the wild-type alcohol gavaged group (Supplemental Figure S4). Accordingly, load- and heart rate–dependent and load- and heart rate–independent indexes of cardiac contractility were not ameliorated in the *Tlr4* knockout mice following alcohol exposure compared with wild-type controls (Supplemental Figure S5).

## Discussion

This study investigated the relationship between single alcohol binge-induced endotoxemia and elevated myocardial levels of the endocannabinoid AEA, which results in CB1R-mediated cardiodepression and blood redistribution following acute alcohol exposure (Figure 7D). The results of the current study offer several interesting findings that merit consideration. At first, acute alcohol-induced blood redistribution contributes to intestinal barrier dysfunction and facilitates the translocation of endotoxins into the circulation. Second, selectively depleting Gram-negative flora from the gut with nonabsorbable antibiotics partially ameliorates intestinal barrier dysfunction, diminishes systemic endotoxemia, and significantly enhances cardiovascular performance after oral alcohol administration by decreasing myocardial AEA formation. Building on previous work describing the detrimental effects of the AEA-CB1R signaling axis in acute alcohol intoxication-induced cardiovascular dysfunction, these findings further emphasize the involvement of AEA-CB1R signaling in the myocardium and vasculature as an important contributor to the adverse cardiovascular effects of alcohol binge.^7^

It was previously shown in a mouse model of acute alcohol administration that binge drinking increases peripheral vasodilation, especially in the acral regions and hind limbs of mice, in a CB1R-mediated manner.^7^ Moreover, others have reported a reduced pulmonary diffusing capacity in human subjects following acute alcohol consumption, possibly because of the alcohol-induced redistribution of blood from the lungs to the periphery. These data suggest that acute alcohol ingestion has a significant impact on vascular tone, promoting vasodilation in certain vascular beds, which subsequently drives blood redistribution.^39^^,^^40^ Additionally, reduced blood volume in the pulmonary capillaries may compromise gas exchange efficiency, affecting oxygenation during increased oxygen demand, such as exercise.^40^, ^41^, ^42^ Previous studies have also shown that gut intraluminal administration of alcohol in dogs can cause mucosal microvascular stasis and intraluminal plasma protein loss.^43^ In line with these findings, a significant reduction in the rate of intestinal perfusion 3 hours after an alcohol binge was observed, as measured by laser speckle contrast imaging (Figure 1, A and B). Thus, alcohol ingestion and subsequent mesenteric microvascular stasis may contribute to a transient increase in both microvascular and epithelial permeability.^44^^,^^45^ Consistent with this, earlier reports have demonstrated a temporal increase in circulating bacteria-derived endotoxin levels following binge drinking.^13^ Even a single episode of binge drinking can rapidly increase serum endotoxin levels because of the translocation of gut-derived bacterial products into the systemic circulation, indicating compromised gut barrier function.^13^^,^^46^ Accordingly, here, it was found that the reduced intestinal perfusion was accompanied by elevated circulating endotoxin levels, implicating alcohol-induced blood flow redistribution as a contributing factor in gut barrier disruption following a single episode of binge drinking (Figure 1C).

Endotoxins (mainly LPS) are found in the outer membranes of Gram-negative bacteria, including *Escherichia coli*.^47^ Alcohol consumption alters bacterial cell membrane integrity, disrupting the function of the outer membrane and, therefore, causing LPS to be shed.^48^ Excessive alcohol drinking also disrupts the intestinal barrier function and facilitates the translocation of bacterial byproducts, including LPS, into the circulation.^46^^,^^49^^,^^50^ Circulating LPS can activate immune cells by interacting with TLRs on their surfaces, triggering inflammatory cascades.^49^^,^^50^ Evidence also suggests that LPS is a potent activator of AEA production in different cell types, including endothelial cells.^29^^,^^51^^,^^52^ Furthermore, oxidative stress has also been linked to the triggering of endocannabinoid formation in tissues, which contributes to the CB1R-dependent cardiodepression, vasodilation, or even vasoconstriction.^21^^,^^22^^,^^24^ This study found that selectively depleting intestinal Gram-negative flora before oral alcohol administration significantly lowered circulating LPS levels and partially ameliorated intestinal leak following a single alcohol binge, as indicated by reduced portal plasma levels of fluorescein isothiocyanate–dextran-4 despite comparable blood alcohol levels among the alcohol-exposed groups (Figure 2B).

To study the implications of alcohol-induced endotoxemia in cardiac AEA formation, a well-established approach was used to selectively deplete intestinal Gram-negative flora using nonabsorbable antibiotics before alcohol gavage. Evidence demonstrates that complete elimination of gut microbiota using broad-spectrum antibiotic treatment is ineffective and potentially harmful in alcohol-related disease: a recent clinical trial using broad-spectrum antibiotics (vancomycin, gentamicin, and meropenem) showed no reduction in serum inflammatory markers or 90-day mortality in patients with alcoholic hepatitis, despite the successful and complete microbiota depletion, suggesting that selective interventions are more beneficial than complete elimination of the intestinal microbiota.^12^^,^^53^ Accordingly, the approach is also known to ameliorate ethanol-induced liver disease by preventing alcohol-induced dysbiosis, stabilizing the gut barrier, and reducing the translocation of bacterial products to the liver.^31^^,^^32^^,^^54^^,^^55^ Consistent with previous findings, no immediate adverse effects were observed from antibiotic pretreatment with respect to liver or kidney injury before alcohol administration (Supplemental Figure S1, A and B). Pretreated animals showed no signs of distress or gastrointestinal upset during daily care. ABx pretreatment of mice, however, significantly altered the composition of their fecal microbiota, demonstrating the expected selective effect on intestinal bacterial communities by reducing the abundance of fecal Gram-negative bacteria, such as the Akkermansiaceae or Muribaculaceae families (Supplemental Figure S1C).

It was also found that acute alcohol intoxication significantly increased the myocardial levels of 4-HNE adducts, reflecting increased oxidative stress (Figure 2, C–E). This finding aligns with established evidence demonstrating a strong positive correlation between alcohol exposure-induced oxidative stress and subsequent increased synthesis of AEA.^56^^,^^57^ Additionally, alcohol-mediated gut barrier dysfunction and subsequently increased circulating LPS levels can independently trigger oxidative stress in cardiac tissue.^58^ On the other hand, AEA can also contribute to the formation of free radicals by activating CB1Rs, potentially generating a feed-forward loop that may worsen cardiac dysfunction after acute alcohol intoxication.^59^^,^^60^ These findings that ABx pretreatment resulted in only a trending reduction in levels of cardiac 4-HNE adducts suggest that gut-derived factors may contribute significantly to alcohol-induced myocardial oxidative stress, beyond the direct effects of ethanol and CB1R activation.

It was also found that acute alcohol exposure significantly altered myocardial gene expression, as shown by increased levels of genes involved in cell death, protein ubiquitination, autophagy, mitogen-activated protein kinase signaling cascade, as well as ethanolamine-containing compound metabolism, along with decreased ATP synthesis and NADH metabolism, compared with the ABx-pretreated alcohol exposed group (Figure 3). Notably, pretreatment with nonabsorbable antibiotics alone had negligible effects on cardiac gene expression profile (Supplemental Figure S2). Additionally, alcohol binge increased the expression of AEA synthetic enzymes, but this effect was absent in the ABx-treated group (Figure 4A). The expression of the major 2-AG synthetic enzymes (*Dagla* and *Daglab*) remained unchanged across the groups. Quantitative real-time PCR analysis also revealed a significant increase in the cardiac expression of key AEA synthetic enzymes (*Napepld*, *Gde1*, and *Abhd4*) in mice exposed to alcohol, whereas ABx pretreatment partially reduced the levels of these genes (Figure 4B). Also, there was no change in the cardiac mRNA levels of fatty acid amide hydrolase (*Faah*), the primary catabolic enzyme of AEA. Liquid chromatography/tandem mass spectrometry revealed increased levels of myocardial AEA in control pretreated animals; however, this was significantly reduced in the ABx pretreated mice following alcohol binge, whereas the levels of the other major endocannabinoid 2-AG remain unchanged (Figure 4). AEA is thought to be synthesized from a precursor, N-arachidonyl phosphatidylethanolamine, in a calcium-dependent manner by the enzyme N-arachidonyl phosphatidylethanolamine phospholipase D. However, two additional calcium-independent pathways also exist for AEA production. The phospholipase C pathway involves phospholipase C and two other enzymes, protein tyrosine phosphatase nonreceptor type 22 and phosphatidylinositol-3,4,5-trisphosphate 5-phosphatase 1.^28^^,^^29^ Another alternative AEA synthetic pathway involves secreted phospholipase A2, α/β-hydrolase domain-containing protein 4 (ABHD4), and glycerophosphodiester phosphodiesterase 1.^61^ Thus, the results indicate that selective depletion of intestinal Gram-negative bacteria and the subsequent decrease in circulating LPS levels following an alcohol binge reduce the expression of key synthetic enzymes that may contribute to increased AEA levels in the heart after acute alcohol exposure.^59^^,^^60^^,^^62^^,^^63^

In this study, it was also found that selective depletion of gut Gram-negative bacteria and the subsequent reduction in myocardial AEA levels were associated with a partially improved cardiac contractility compared with the control alcohol group (Figures 5 and 6). These findings align with prior research demonstrating the positive effects of inhibiting CB1R signaling in the cardiovascular system after acute alcohol exposure, resulting in improved cardiac performance.^7^^,^^18^ It was previously shown that the activation of the myocardial AEA-CB1R signaling axis may also decrease LV intrinsic contractility, as determined by evaluating load- and heart rate–independent indexes of LV performance.^7^^,^^22^^,^^60^^,^^62^ In line with these findings, a partially improved intrinsic contractility in alcohol-gavaged mice with prior ABx treatment was documented (Figure 6), suggesting that selective depletion of intestinal Gram-negative bacteria and the subsequent reduction in circulating LPS levels contribute to a decreased myocardial AEA formation, resulting in better contractility following acute alcohol intoxication.

It has been previously reported that high doses of acute alcohol induce hypotension, which decreases total peripheral resistance and cardiac function.^7^^,^^64^ Consistent with these findings, a significant reduction in mean arterial blood pressure and total peripheral resistance in alcohol-exposed, control-pretreated mice was demonstrated. In contrast, ABx-pretreated mice exposed to alcohol showed significantly higher mean arterial blood pressure and subsequently increased total peripheral resistance compared with the control alcohol group (Figure 7A), which coincided with improved peripheral vascular tone (Figure 7, B and C). Furthermore, antibiotic pretreatment before alcohol administration significantly increased blood flow through the superior mesenteric artery 3 hours after alcohol administration, possibly because of enhanced cardiac function; however, no significant difference was noted in the rate of intestinal microcirculation among the alcohol-exposed groups, suggesting other underlying mechanisms may be implicated in impairing mesenteric microcirculation following binge drinking (Supplemental Figure S3).

The contribution of TLR4 activation to myocardial AEA formation in the context of binge alcohol-induced endotoxemia using *Tlr4*^*–*^*/*^*–*^ mice was also investigated. Although serum alcohol and circulating endotoxin levels were comparable between wild-type and *Tlr4*^*–*^*/*^*–*^ groups 3 hours after the acute alcohol exposure, both genotypes showed similarly elevated cardiac AEA levels (Supplemental Figure S4). Accordingly, similar impairment in left ventricular contractile function among the alcohol gavaged groups was found (Supplemental Figure S5). These results suggest that although TLR4 signaling plays a well-established role in cardiac dysfunction, alternative molecular pathways may be responsible for alcohol-induced myocardial AEA formation and the associated impaired cardiac contractility. Similar to TLR4, TLR2 is expressed in multiple cell types within the cardiovascular system, including cardiomyocytes, endothelial cells, and tissue-resident macrophages. Recent research indicates that activation of TLR2 in the myocardium enhances inflammatory responses and plays a role in adverse cardiac remodeling and dysfunction.^65^^,^^66^ Therefore, following acute alcohol intoxication, LPS may trigger cardiovascular dysfunction not only through TLR4 but also via TLR2-mediated signaling pathways, potentially worsening myocardial injury and impairing cardiac function. In addition to LPS, other Gram-negative, bacterial-derived products, including outer membrane vesicles, metabolites, and proteoglycans, may contribute to myocardial AEA formation and subsequent cardiodepression following acute alcohol exposure.^67^^,^^68^

## Conclusions

This study reveals an important crosstalk between intestinal barrier dysfunction and cardiovascular dysfunction following an acute alcohol binge, with AEA as a crucial connecting factor. The concurrent reduction in intestinal perfusion and elevation of circulating LPS levels at 3 hours after alcohol gavage underlines the rapid yet transient disruption of gut barrier integrity induced by alcohol. In mice, antibiotic pretreatment effectively attenuated the alcohol binge-induced intestinal barrier dysfunction and subsequent endotoxemia, which was paralleled by a significant reduction in myocardial AEA levels. These findings suggest that selective depletion of intestinal Gram-negative bacteria alleviates alcohol-induced cardiovascular dysfunction, at least partially through modulation of AEA signaling. Although it is important to emphasize that antibiotic pretreatment is not promoted or recommended as a preventive measure before binge alcohol drinking, this improvement in hemodynamics following an alcohol binge highlights the therapeutic potential of targeting the gut microbiome and endocannabinoid system to mitigate alcohol-related cardiovascular complications, highlighting the importance of the gut-heart axis in the context of binge drinking.

Several limitations should be acknowledged in the present study. Although the findings demonstrate the protective effects of targeted depletion of intestinal Gram-negative bacteria, the current approach does not identify specific bacterial taxa or specific signaling pathways responsible for gut barrier dysfunction, nor their exact roles in cardiovascular endocannabinoid signaling. Future research will be crucial for determining the specific factors contributing to gut barrier integrity and cardiac endocannabinoid-related pathways, which may facilitate the development of more targeted therapeutic interventions.

## Disclosure Statement

None declared.

## Supplemental Data

Supplemental Figure S1 Pretreatment with antibiotics (ABx) effectively depletes intestinal Gram-negative bacteria in mice without causing liver and kidney damage. **A:** Body weight changes in mice during oral control (ctrl) and ABx pretreatment (ABx). *P* value indicates main effects of ABx on body weight change over the course of 13 days (two-way repeated measures analysis of variance with Bonferroni multiple comparison test). **B:** Serum levels of alanine aminotransferase (ALT) and blood urea nitrogen in ctrl and ABx mice. The *t*-test was used. **C:** Relative abundance plot showing different fecal bacterial families within each sample before and after antibiotic pretreatment. Data are presented as group means ± SEM (**A**) or individual values with group means ± SEM (**B**). *n* = 50 to 52 (**A**); *n* = 7 (**B**). ∗*P* < 0.05.

Supplemental Figure S2 Transcriptomics data analysis. **A:** Principal component (PC) analysis of the cardiac transcriptome across experimental groups. **B:** The number of myocardial differentially expressed genes (DEGs; adjusted *P* < 0.05) showing transcriptional differences between mice gavaged either with saline or alcohol [ethanol (EtOH)], with or without antibiotic pretreatment (ABx or ctrl, respectively).

Supplemental Figure S3 Selective depletion of gut Gram-negative bacteria does not mitigate binge alcohol-induced impairment of intestinal microcirculation. **A:** Representative images of the intestinal microcirculation measured in mice 3 hours after the administration of saline or alcohol [ethanol (EtOH)], with or without antibiotic pretreatment (ABx or ctrl, respectively). Red indicates higher intensity, whereas blue represents lower rate of microcirculation. **B:** The results of hind limb microcirculation in mice 3 hours after the gavage saline across the experimental groups. **C:** Blood perfusion through the superior mesenteric artery measured in mice 3 hours after administering saline or alcohol (ethanol), with or without antibiotic pretreatment (ABx or ctrl, respectively). Data are presented as individual values with group means ± SEM (**B** and **C**). *n* = 8 to 13 (**B** and **C**). ∗*P* < 0.05 versus corresponding saline group; ^#^*P* < 0.05 versus ctrl ethanol group (two-way analysis of variance, Bonferroni multiple comparison test). AU, arbitrary unit.

Supplemental Figure S4 *Tlr4*^*–/–*^ mice exhibit similar increases in circulating lipopolysaccharide and myocardial anandamide (AEA) levels compared with their wild-type (WT) counterparts following acute alcohol intoxication. **A****:** Serum alcohol and endotoxin levels measured 3 hours after oral administration of saline or alcohol [ethanol (EtOH)] in WT and *Tlr4*^*–/–*^ mice. **B:** Myocardial levels of AEA 3 hours after the oral administration of saline or alcohol (ethanol) in WT and *Tlr4*^*–/–*^ mice. Data are presented as individual values with group means ± SEM (**A** and **B**). *n* = 4 to 9 (**A** and **B**). ∗*P* < 0.05 versus corresponding saline group (two-way analysis of variance, Bonferroni multiple comparison test). EU, endotoxin unit.

Supplemental Figure S5 *Tlr4*^*–/–*^ mice show similar impairments in myocardial contractility compared with their wild-type (WT) counterparts following acute alcohol intoxication. **A:** Systolic indexes of left ventricular (LV) performance [maximal slope of pressure increment (dP/dt_max_), end-systolic pressure, stroke work, stroke volume, cardiac output, and ejection fraction] in WT and *Tlr4*^*–/–*^ mice 3 hours after the administration of saline or alcohol [ethanol (EtOH)]. **B:** Load- and heart rate–independent indexes of LV performance [end-systolic elastance (Ees), preload-recruitable stroke work (PRSW), and the dP/dt_max_–end-diastolic volume (dP/dt_max_-EDV) relation] in WT and *Tlr4*^*–/–*^ mice 3 hours after the administration of saline or alcohol (ethanol). Data are presented as individual values with group means ± SEM (**A** and **B**). *n* = 5 to 8 (**A** and **B**). ∗*P* < 0.05 versus corresponding saline group (two-way analysis of variance, Bonferroni multiple comparison test).
