# Supplementary figures and images for: Selective Depletion of Gut Gram-Negative Bacteria Attenuates Alcohol Binge-Induced Cardiovascular Dysfunction by Lowering Cardiac Anandamide Levels
Source: Am J Pathol. 2025 Nov 6;196(1):192–208. doi: 10.1016/j.ajpath.2025.10.010 (PMC12799518; doi:10.1016/j.ajpath.2025.10.010)

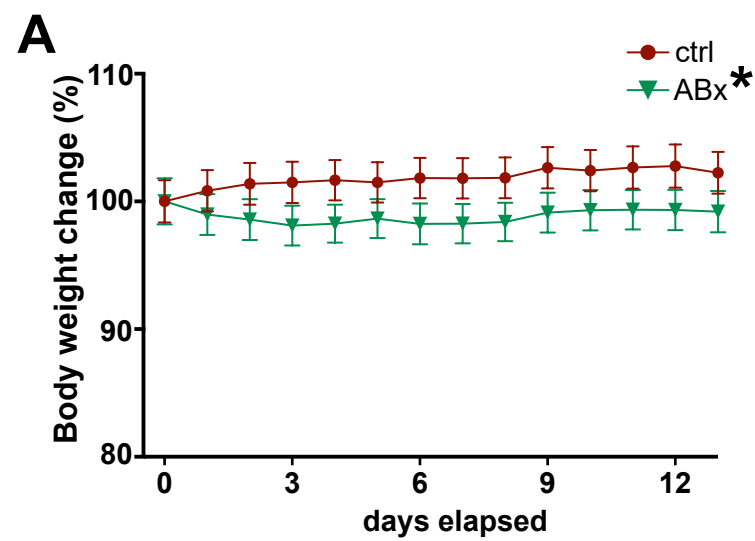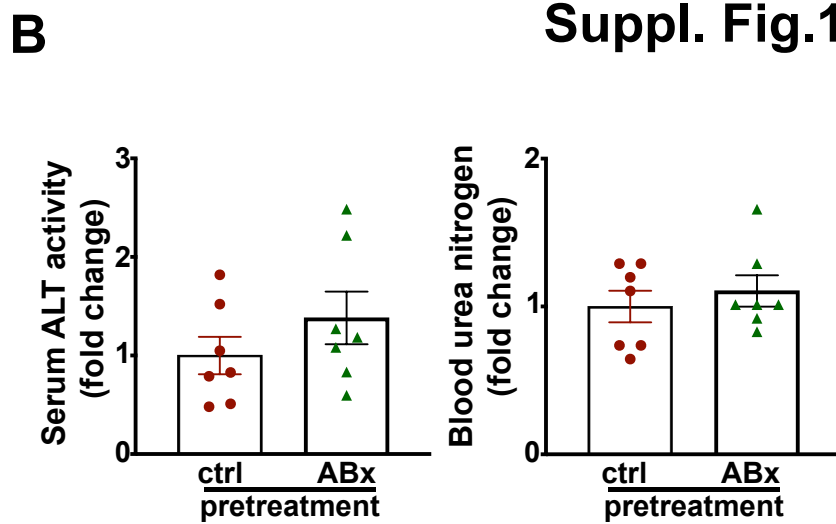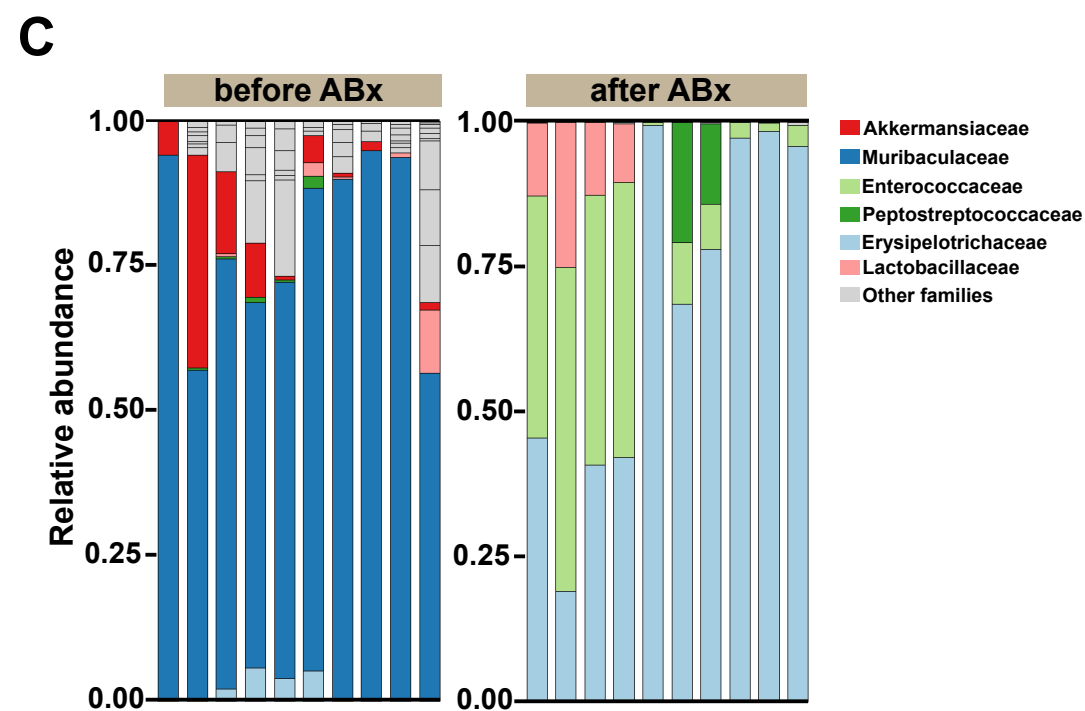

Supplement: Supplemental Figure S1 — Pretreatment with antibiotics (ABx) effectively depletes intestinal Gram-negative bacteria in mice without causing liver and kidney damage. A: Body weight changes in mice during oral control (ctrl) and ABx pretreatment (ABx). P value indicates main effects of ABx on body weight change over the course of 13 days (two-way repeated measures analysis of variance with Bonferroni multiple comparison test). B: Serum levels of alanine aminotransferase (ALT) and blood urea nitrogen in ctrl and ABx mice. The t-test was used. C: Relative abundance plot showing different fecal bacterial families within each sample before and after antibiotic pretreatment. Data are presented as group means ± SEM (A) or individual values with group means ± SEM (B). n = 50 to 52 (A); n = 7 (B). ∗P < 0.05. [file mmc1.pdf]

**A**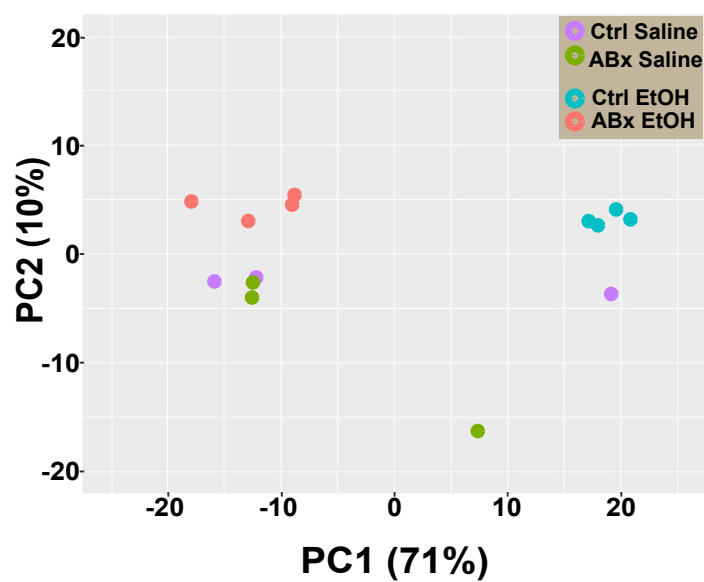**B****Suppl. Fig.2**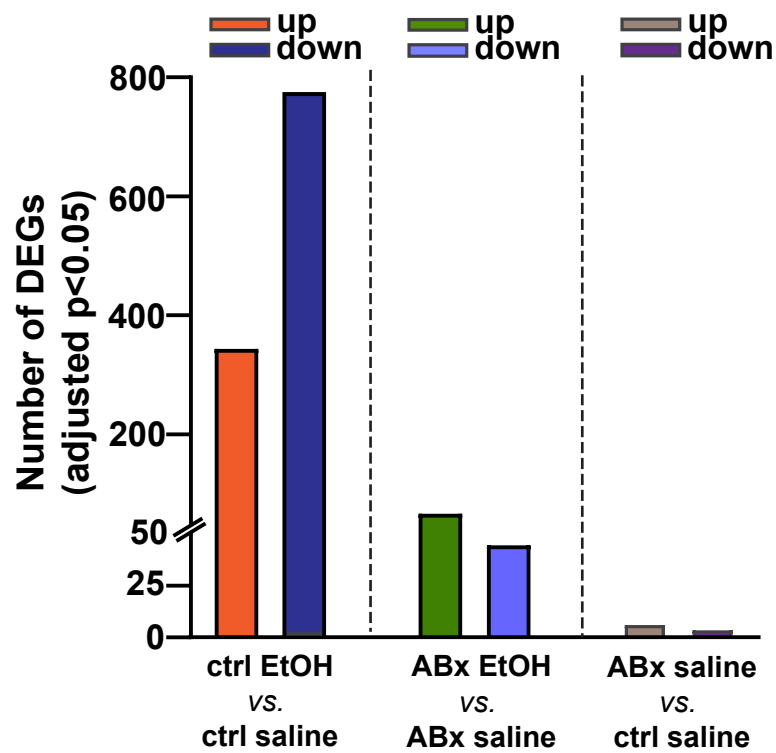

Supplement: Supplemental Figure S2 — Transcriptomics data analysis. A: Principal component (PC) analysis of the cardiac transcriptome across experimental groups. B: The number of myocardial differentially expressed genes (DEGs; adjusted P < 0.05) showing transcriptional differences between mice gavaged either with saline or alcohol [ethanol (EtOH)], with or without antibiotic pretreatment (ABx or ctrl, respectively). [file mmc2.pdf]

**A**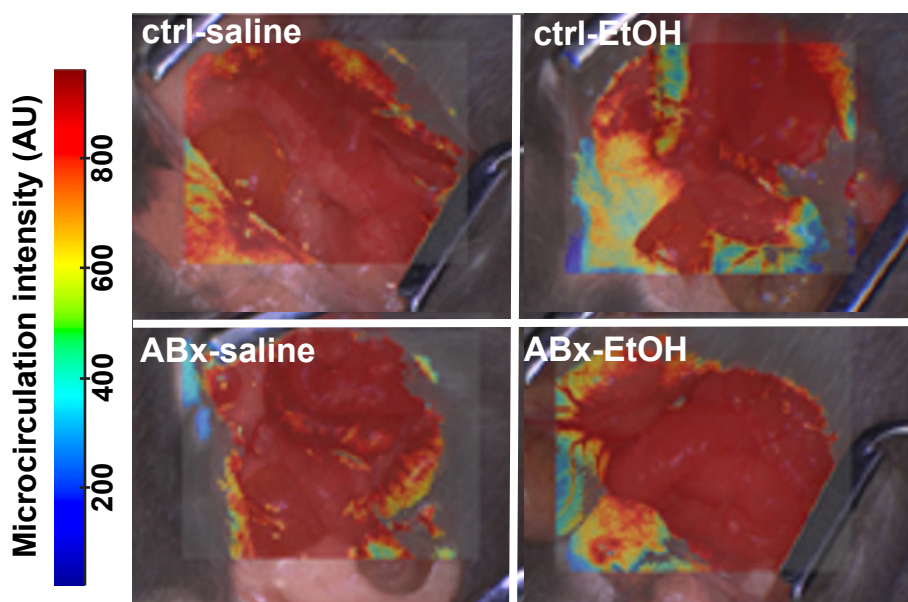**B****Suppl. Fig.3**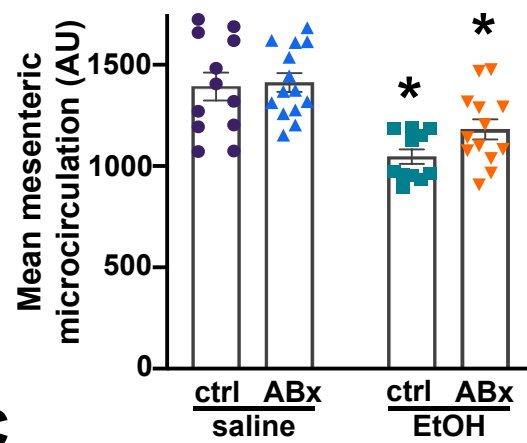**C**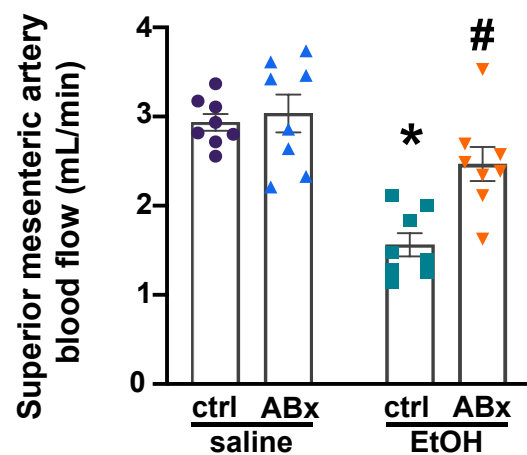

Supplement: Supplemental Figure S3 — Selective depletion of gut Gram-negative bacteria does not mitigate binge alcohol-induced impairment of intestinal microcirculation. A: Representative images of the intestinal microcirculation measured in mice 3 hours after the administration of saline or alcohol [ethanol (EtOH)], with or without antibiotic pretreatment (ABx or ctrl, respectively). Red indicates higher intensity, whereas blue represents lower rate of microcirculation. B: The results of hind limb microcirculation in mice 3 hours after the gavage saline across the experimental groups. C: Blood perfusion through the superior mesenteric artery measured in mice 3 hours after administering saline or alcohol (ethanol), with or without antibiotic pretreatment (ABx or ctrl, respectively). Data are presented as individual values with group means ± SEM (B and C). n = 8 to 13 (B and C). ∗P < 0.05 versus corresponding saline group; #P < 0.05 versus ctrl ethanol group (two-way analysis of variance, Bonferroni multiple comparison test). AU, arbitrary unit. [file mmc3.pdf]

**A**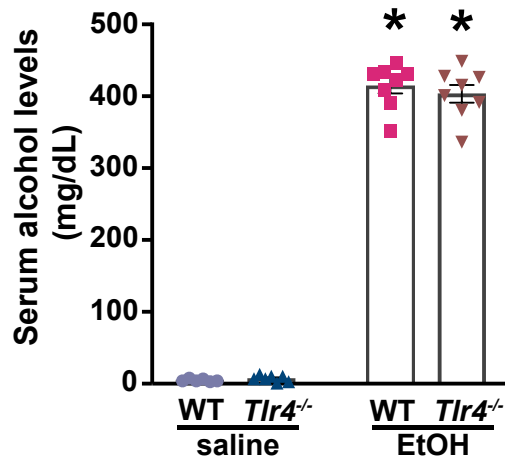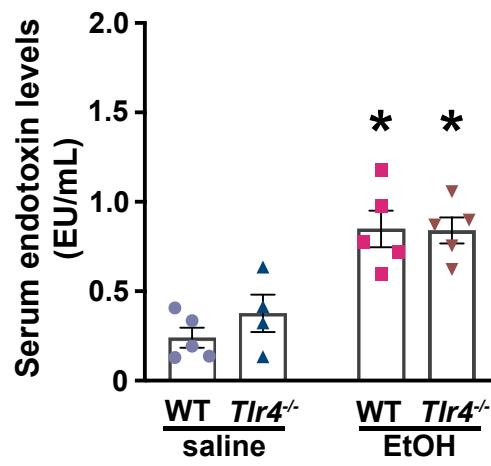**B**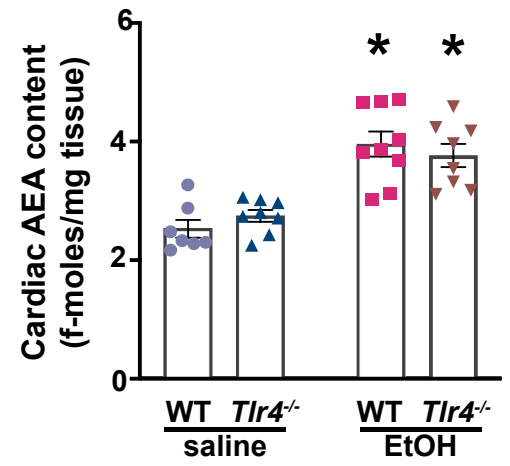

Supplement: Supplemental Figure S4 — Tlr4–/– mice exhibit similar increases in circulating lipopolysaccharide and myocardial anandamide (AEA) levels compared with their wild-type (WT) counterparts following acute alcohol intoxication. A: Serum alcohol and endotoxin levels measured 3 hours after oral administration of saline or alcohol [ethanol (EtOH)] in WT and Tlr4–/– mice. B: Myocardial levels of AEA 3 hours after the oral administration of saline or alcohol (ethanol) in WT and Tlr4–/– mice. Data are presented as individual values with group means ± SEM (A and B). n = 4 to 9 (A and B). ∗P < 0.05 versus corresponding saline group (two-way analysis of variance, Bonferroni multiple comparison test). EU, endotoxin unit. [file mmc4.pdf]

**A**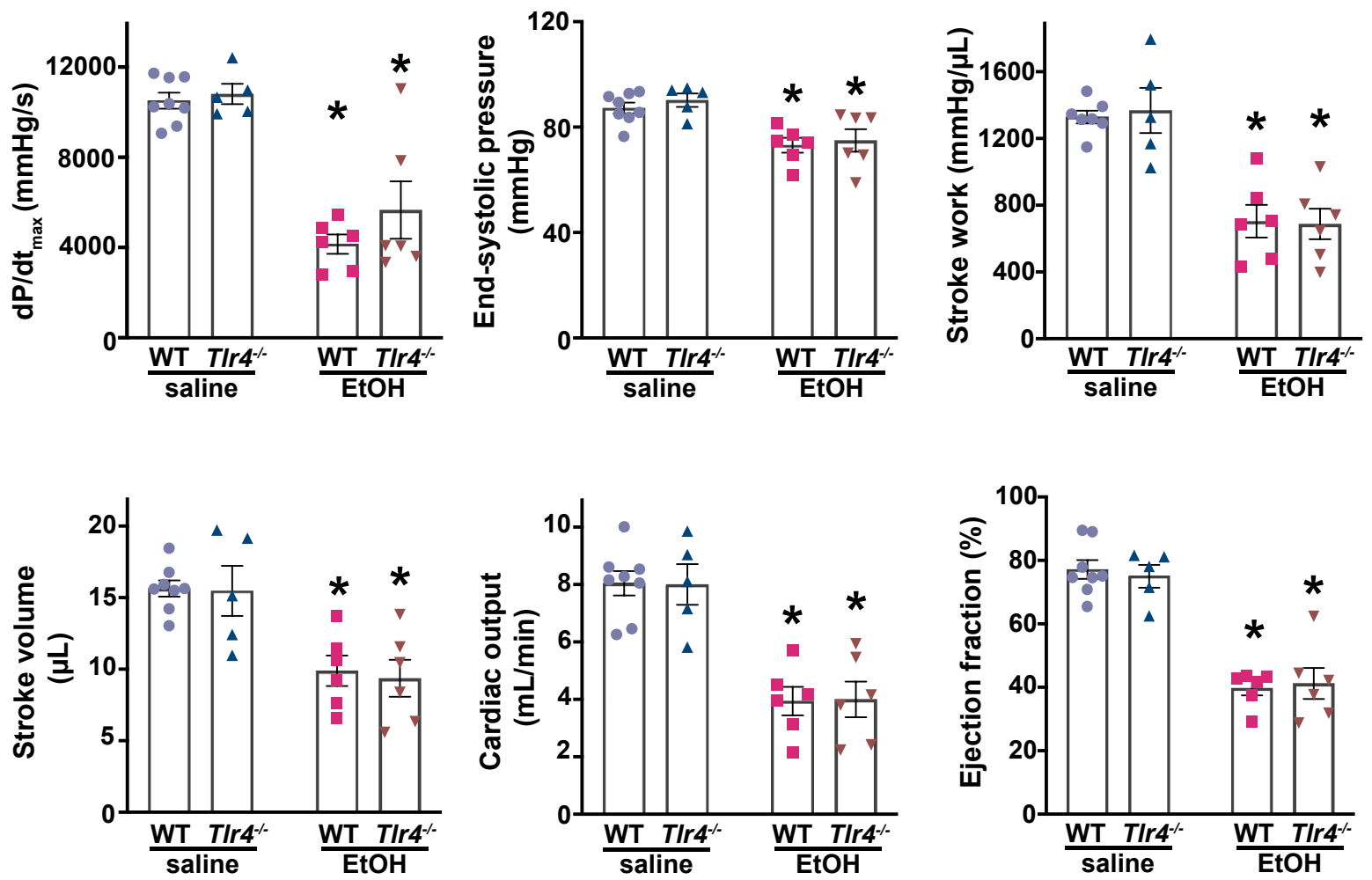**B**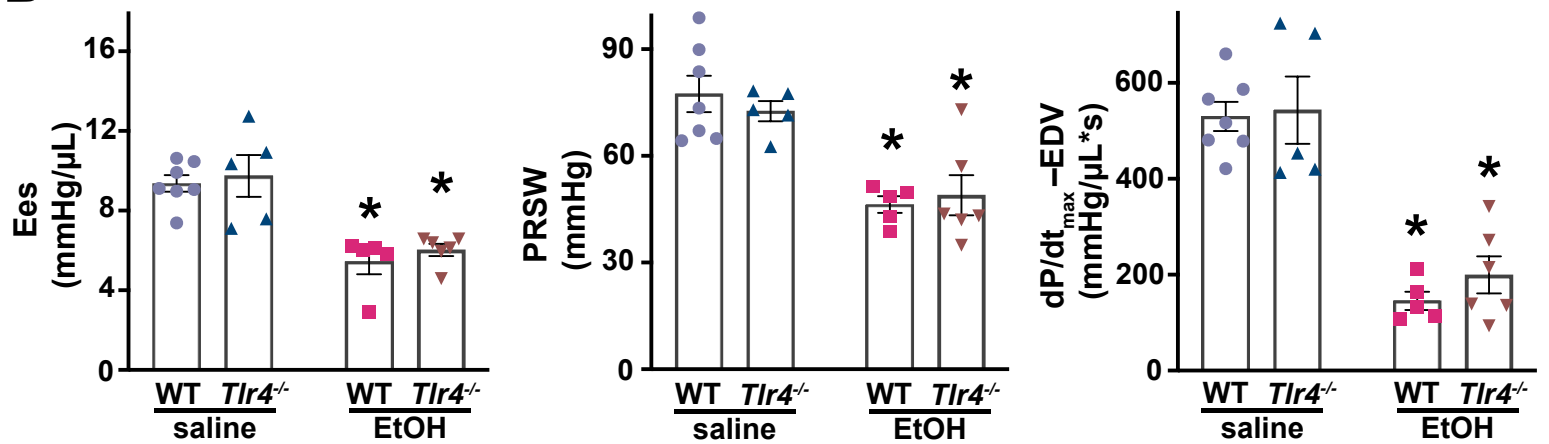

Supplement: Supplemental Figure S5 — Tlr4–/– mice show similar impairments in myocardial contractility compared with their wild-type (WT) counterparts following acute alcohol intoxication. A: Systolic indexes of left ventricular (LV) performance [maximal slope of pressure increment (dP/dtmax), end-systolic pressure, stroke work, stroke volume, cardiac output, and ejection fraction] in WT and Tlr4–/– mice 3 hours after the administration of saline or alcohol [ethanol (EtOH)]. B: Load- and heart rate–independent indexes of LV performance [end-systolic elastance (Ees), preload-recruitable stroke work (PRSW), and the dP/dtmax–end-diastolic volume (dP/dtmax-EDV) relation] in WT and Tlr4–/– mice 3 hours after the administration of saline or alcohol (ethanol). Data are presented as individual values with group means ± SEM (A and B). n = 5 to 8 (A and B). ∗P < 0.05 versus corresponding saline group (two-way analysis of variance, Bonferroni multiple comparison test). [file mmc5.pdf]
